# Are pNF-H, IL-6, BDNF, and NSP Reliable Biomarkers of Cognitive Function in Prostate Cancer Patients?

**DOI:** 10.3390/ijms262010202

**Published:** 2025-10-20

**Authors:** Alicja Popiołek, Bartosz Brzoszczyk, Alina Borkowska, Piotr Jarzemski, Mariusz Kozakiewicz, Adam Szelągowski, Maciej Bieliński

**Affiliations:** 1 Department of Clinical Neuropsychology, Nicolaus Copernicus University in Toruń, Collegium Medicum in Bydgoszcz, 87-100 Toruń, Poland; alab@cm.umk.pl (A.B.); bielinskim@gmail.com (M.B.); 2 Department of Internal Diseases, Jan Biziel University Hospital No. 2 in Bydgoszcz, 85-168 Bydgoszcz, Poland; 3 Department and Clinic of Urology, Jan Biziel University Hospital No. 2 in Bydgoszcz, Nicolaus Copernicus University in Toruń, Collegium Medicum in Bydgoszcz, 87-100 Toruń, Poland; bartosz.brzoszczyk@gmail.com (B.B.); piotr@jarzemski.pl (P.J.); 4 Faculty of Medicine, The Mazovian University in Płock, 09-402 Płock, Poland; 5 Department of Geriatrics, Nicolaus Copernicus University in Toruń, Collegium Medicum in Bydgoszcz, 87-100 Toruń, Poland; markoz@cm.umk.pl (M.K.); aszelagowski@doktorant.umk.pl (A.S.)

**Keywords:** prostate cancer, cognitive function, biochemical markers, Il-6, BDNF, pNF-H, NSP

## Abstract

Cognitive decline can result from various factors, including direct neurotoxic injury, brain tissue damage, inflammation, and disruptions in coagulation and fibrinolysis. This study aimed to examine the relationship between biochemical markers associated with cognitive function and cognitive performance in men with prostate cancer (PC) following radical prostatectomy. Participants underwent a comprehensive evaluation, including clinical assessments (demographic information, medical history, PC progression, and complications such as erectile dysfunction [IIEF-5] and urinary incontinence [ICIQ-UI]), biochemical testing (testosterone, prostate-specific antigen, phosphorylated neurofilament heavy chain [pNF-H], brain-derived neurotrophic factor [BDNF], neuroserpin [NSP], and interleukin-6 [IL-6]), and neuropsychological assessment of cognitive functions. Statistical analysis revealed significant positive correlations between BDNF and NSP levels and performance on delayed memory tasks, specifically the number of correct responses. No other significant associations were found between protein biomarkers and cognitive test outcomes. These findings suggest that the relationship between biochemical markers and cognitive function is complex. However, BDNF and NSP may serve as potential biomarkers for delayed memory impairment in men post-prostatectomy.

## 1. Introduction

Cognitive decline can arise from a range of underlying factors. Multiple mechanisms have been proposed to contribute to cognitive dysfunction, including inflammation, coagulation, and fibrinolysis, as well as direct neurotoxic injury and brain tissue damage [1]. These mechanisms are observed to varying extents across a range of somatic diseases [2,3], and are notably present in cancer and its associated treatments. This may help explain the more rapid onset of cognitive impairment in individuals with cancer [4]. One of the malignancy linked to cognitive decline is prostate cancer (PC). This might be connected to the disease itself and the treatment given [5].

### 1.1. Inflammation

Typically, inflammation is a protective response that aids healing. However, chronic inflammation might damage tissues, potentially causing somatic and neurodegenerative disorders. The link between inflammation and numerous diseases is under increased scrutiny by researchers. Notably, inflammation is implicated in approximately 20% of all cancers [6]. It has been linked to cancer development in various organs, including the stomach, liver, and large intestine [7]. A growing body of evidence points also to chronic inflammation’s role in PC development and progression [8,9]. It has been hypothesized that persistent neuroinflammation—initiated by the innate immune response, deoxyribonucleic acid damage, or endothelial dysfunction—plays a central role in the pathogenesis of cognitive decline [10]. Furthermore, inflammatory infiltration within prostate tumors has been associated with poorer prognosis and reduced therapeutic efficacy [11].

Sfanos and De Marzo’s review emphasizes the significance of interleukin 6 (Il-6), a cytokine of interest in PC, especially its contribution to advancement and disease progression [12]. Similar observations have been made by other scientists who note Il-6 systemic levels’ association with the metastatic process and aggressive PC phenotype [8,13,14].

### 1.2. Coagulation and Fibrinolysis

Disorders in the coagulation and fibrinolysis systems are common features of malignancies [15]. Solid tumors can disrupt these systems, leading to blood coagulation complications such as thromboembolic events and severe bleeding [16]. Research has established a link between levels of specific components—such as von Willebrand factor, D-dimers, and neuroserpin (NSP)—and cognitive decline [17,18]. However, the specificity of these markers remains relatively low. NSP, a serine protease inhibitor, was found during a search for proteins involved in neuronal axon growth and synapse formation [19]. It is a modulator of synaptic plasticity that plays an important role in learning and memory [18]. To assess NSP’s role in brain development, Hermann and colleagues studied the hippocampi of mice lacking NSP. They found NSP essential for creating the correct neuronal network, normal synaptic plasticity, regulating appropriate cognitive, emotional, and social behavior in mice [20]. Other researchers confirmed NSP’s role in synaptogenesis and synaptic plasticity through behavioral studies of transgenic mice, suggesting a potential cellular mechanism for its cognitive effects [21].

### 1.3. Brain Tissue Damage

Cancer can also directly impact brain tissue [22], often by damaging the blood–brain barrier, either spontaneously or as a consequence of treatment [23]. A frequent result of such damage is the release of brain injury-associated proteins.

The phosphorylated form of the high-molecular-weight neurofilament heavy subunit (pNF-H) is a key structural protein in axons. Its serum level serves as a biomarker for axonal injury [24]. Elevated levels are reported in the literature following brain injury [25], traumatic spinal cord injury [26], burns [27], and various neurological diseases [28]. There is no direct correlation between its cerebrospinal fluid and blood levels [29]. Increased blood levels following injury predict patient outcomes. This is helpful in evaluating neuropathology after traumatic brain injury [30], axonal repair [31], post-injury epilepsy [32], vegetative states, mortality [33,34], and cognitive function [35]. According to a study from Japan, pNf-H levels are associated with serum biomarkers of compromised blood–brain barrier disruption [36]. Prior studies have measured this protein in cancer patients who underwent surgery [37] or chemotherapy [38]. Chemotherapy patients with breast cancer showed higher serum pNf-H levels with cumulative doses, indicating its possible use as a neural damage biomarker after chemotherapy [38].

### 1.4. Other Biomarkers of Cognitive Decline

Based on published research, BDNF and its receptor levels are crucial regulators in the male genitourinary system, impacting diseases like PC, benign prostate hyperplasia, infertility, diabetic erectile dysfunction, penile sclerosis, and bladder fibrosis [39]. New nerve growth in the prostate tumor microenvironment is critical for PC progression [40]. This is probably why BDNF levels could be associated with the origin and advancement of PC [41].

BDNF is also a key biomarker for cognitive functions. This neurotrophin protein, secreted by nerve cells, influences neuronal growth, function, memory, and learning. Cognitive symptoms are linked to its levels in the central nervous system (CNS) and blood. Researchers propose that genetics may play a role in the cognitive decline observed in oncology patients [42,43]. A study indicates that the BDNF rs6265 Met/Met genotype elevates the likelihood of cognitive problems in those with breast cancer [44]. Genetic predisposition, combined with negative environmental influences, can raise the probability of these dysfunctions in individuals [43], while favorable factors can reduce this risk [45].

While these markers have been studied in relation to various diseases, including cancer, research specifically investigating brain tissue damage markers in PC patients remains limited.

The identification of reliable biomarkers of cognitive decline in oncology populations—specifically among men with PC—could facilitate the recognition of subgroups at increased risk of such dysfunctions. This, in turn, may open the way for preventive strategies, whereby patients could benefit from interventions such as cognitive training, pharmacological treatments, or tailored supportive care, ultimately helping to mitigate the impact of cognitive decline on quality of life [46]. Nonetheless, further research in this area remains necessary. Accordingly, the aim of our study was restricted to evaluating the validity of cognitive function biomarkers in patients with PC.

Emerging evidence points to several potential plasma biomarkers for cognitive impairment in cancer patients [10]. These include inflammatory mediators (e.g., Il-6), components of the coagulation and fibrinolysis systems (e.g., NSP), markers of brain tissue damage (pNF-H), and other biochemical markers (BDNF) [1]. Studies have shown the presence of these markers across various cancers, suggesting potential pathways leading to cognitive dysfunction [38,47].

This study aims to explore potential associations between biochemical markers related to cognitive dysfunction and cognitive performance in men with PC after radical prostatectomy.

## 2. Results

Demographic, clinical, and cognitive data can be found in Table 1.

Patients in Grades 4 and 5 who had received additional adjuvant therapy (including chemotherapy) were excluded from further analyses.

Comparative analyses of the tested protein levels were conducted across subgroups defined by smoking status, physical activity, and body weight (Table 2). No significant differences were observed, except for BDNF, which showed lower levels in the smokers’ group; however, this result only approached statistical significance.

The levels of the tested proteins were correlated with various clinical parameters and cognitive test scores (Table 3). A significant negative correlation between NSP levels and age was observed. In subsequent analyses, only significant positive correlations were identified between BDNF and NSP levels and the number of correct responses in the delayed memory test. No other significant correlations were found between protein levels and the results of the Neurotest computer battery assessments.

To validate the accuracy of the delayed memory test (Table 4), a control analysis examining the correlation between test scores and age was conducted, revealing a significant decline in performance with increasing age. Additionally, responses in the delayed memory test showed significant correlations with other assessments, including the short-term memory and visual working memory tests. Reaction time results in the simple reaction time test also demonstrated meaningful correlations; higher VMDT scores were associated with faster reaction times.

**Figure 1:**
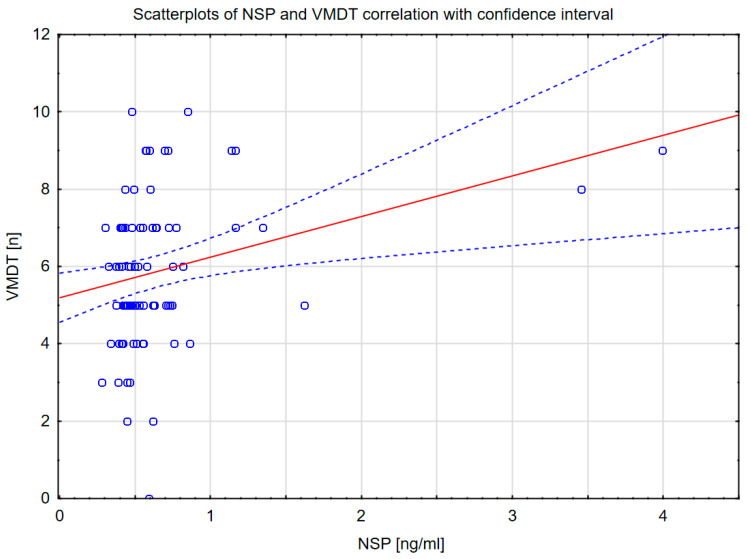
Scatterplots of NSP and VMDT correlation with confidence interval. Abbreviations: NSP, neuroserpin; VMDT, verbal memory delayed test.

Scatterplots of NSP and VMDT correlation with confidence interval was performed and is presented in Figure 1.

Furthermore, qualitative analyses were performed to evaluate protein levels in relation to clinical factors such as hypertension, diabetes, myocardial infarction, stroke, obesity, smoking status, physical activity, and PC staging and grading (TNM, GS, GG) as well as medications used. No significant differences were detected. Correlation analyses between protein levels and clinical and biochemical parameters—including BMI, erectile function, taking erection medications, hormone therapy, time since procedure, PSA, testosterone levels, and CRP—and also yielded no significant findings.

## 3. Discussion

### 3.1. Il-6

Cognitive decline is known to be associated with chronic inflammation [48]. At the molecular level, the role of Il-6 is highlighted here [13]. It regulates CNS pathways essential for cognitive function, controlling various neuronal and synaptic processes including transmission and plasticity, crucial for memory and learning [49]. The relationship between Il-6 levels and cognitive function has been noted in numerous studies across different health states, spanning from diseases like rheumatoid arthritis [50], type 2 diabetes [51], and schizophrenia [52] to healthy populations [53]. However, our studies failed to reveal the expected correlation between cognitive functions and Il-6 levels (Table 3). Tegeler et al. [54] found no correlation between inflammatory markers and verbal episodic memory in their study of elderly German adults. However, they found that higher levels of Il-6, interleukin-10, and CRP negatively correlate with a composite score of executive function and processing speed [54]. Studies conducted in large prospective cohorts confirm correlations between higher plasma Il-6 levels and worse cognitive performance in numeric memory, prospective memory, reaction time tests and pair matching, as well as correlations with structural changes in the brain and increased risk of dementia [55]. The lack of correlations in our studies is intriguing, despite convincing data from the literature suggesting that such relationships could occur. The hypothesis that PC-associated cognitive dysfunction could have a different pathophysiology than the inflammatory pathway is therefore supported by this.

### 3.2. NSP

The data from the literature raise conflicting evidence on whether NSP is neuroprotective (since it is responsible for neuroplasticity, as mentioned in the introduction) or involved in dementia progression [56]. In our study, higher level of NSP correlate with better ability of delayed memory (Table 3). Other studies have revealed a strong link between NSP levels and cognitive function in people with Alzheimer’s disease (AD) [56,57] and Lewy body diseases [58]. Barba et al. noticed that NSP level may show a complex relationship with cognitive decline when reduced and with AD pathology when increased [58].

Research into NSP’s role in cancer is expanding and improving [59]. The expression of NSP is found in cancer cells; this includes metastatic cells [60]. Its function is thought to involve decreasing protective plasmin, thereby promoting brain metastases [61]. Elevated NSP levels suppress tissue plasminogen activator’s neuroprotective effects and plasmin formation, thus decreasing beta-amyloid (Aβ) clearance. Neuronal clearance pathway malfunction causes Aβ to accumulate [56,62,63].

Research shows a connection between higher NSP levels, poorer prognoses, and potential chemotherapy resistance in breast cancer patients, supporting this claim [64]. Notably, NSP genes show high expression in PC, correlating with its progression and severity [65].

However, the literature lacks data elucidating the mechanism underlying the association between higher NSP levels and better delayed memory in patients with prostate cancer. Based on evidence of its neuroprotective effects in other populations, it can be hypothesized that, despite its association with poorer prognosis, NSP may exert protective effects on hippocampal neurons, reduce oxidative stress, and enhance synaptic plasticity.

### 3.3. pNf-H

Interestingly, our study found no correlation between pNf-H and any of the assessed cognitive domains (Table 3). The study by Trifilio et al. suggests that short-term increases in serum pNF-H could be associated with worse cognitive performance [35]. A mouse model of traumatic brain injury revealed cognitive deficits (spatial and visual memory) after injury, indicated by pNF-H expression in the cortex and hippocampus [31].

American scientists conducted an interesting study assessing this protein’s profile in cerebrospinal fluid and blood after brain injury, both acutely and long-term. They found that serum pNF-H levels peaked approximately between days 20 and 30, then fell. Notably, its persistent post-injury levels also predicted prognosis [66]. The crucial aspect is the time elapsed between axon injury and the onset of dysfunction. Neuropsychological testing of patients varied across prior studies in terms of timing, yet a substantial number of tests happened within the one-year timeframe after chemotherapy [38]. This may also be the reason why the expected relationships were not observed in our studies—considerable delay existed between prostate treatment and cognitive function assessment (the median is 1.5 years after prostatectomy).

### 3.4. BDNF

Studies have also shown a link between low BDNF levels and cognitive decline in people with cancer [67]. This applied to patients receiving chemotherapy, radiotherapy, and regardless of oncological treatment [43,68,69]. Our study reveal a positive correlation between BDNF and better performance on a delayed memory test (Table 3). It is interesting that delayed memory showed significant correlations with both BDNF and NSP in the present study. Delayed memory impairment is often the first to be identified in research and is a key predictor of dementia [70,71,72]. Furthermore, our analyses showed significant correlations between delayed memory test responses and reaction time, short-term memory, and visual working memory performance (Table 4). This suggests its potential relevance to other cognitive disorders.

Like other proteins, BDNF levels showed no significant change with varying physical activity or body mass. Nevertheless, the smokers’ group had lower levels, but this result only approached statistical significance (Table 2). The literature on this topic presents conflicting data [73].

The prostate is a major source of nerve growth factors, including BDNF, outside the nervous system [74]. In PC, these factors are often overexpressed, correlating with lymph node metastasis and advanced disease. In vitro, recombinant BDNF promotes cancer cell migration and invasion [41]. Tumor-associated neurogenesis contributes to disease progression: adrenergic signaling from autonomic nerves supports early tumor growth, while parasympathetic cholinergic signaling facilitates dissemination and metastasis [75]. Interestingly, similar to NSP, BDNF may also support cognitive function. However, tumor microenvironment levels do not necessarily reflect circulating or CNS concentrations, and the relationship between these compartments remains poorly understood. To date, the few studies examining this phenomenon at the molecular level have not demonstrated a clear association [76].

## 4. Materials and Methods

This cross-sectional observational, single-center study was carried out in the urological outpatient department of the Jan Biziel University Hospital No 2 in Bydgoszcz, Poland. The study included 100 patients, Caucasian, diagnosed with prostate adenocarcinoma, treated surgically with radical prostatectomy. The median age of the participants was 66 years (range 50–77 years). Prior to inclusion, all participants gave their written informed consent. The following inclusion criteria were adopted: ability to understand the purpose of the study and willingness to participate, lack of incapacitation, and histopathologically confirmed prostate adenocarcinoma treated with radical prostatectomy. We excluded participants with dementia or other neurodegenerative diseases, diagnosed mental illness, and unstable medical conditions including active infections.

All patients underwent an assessment of clinical status and an assessment of biochemical parameters as well as neuropsychological functions.

The clinical assessment consisted of a patient interview and physical examination. The patient interview included an evaluation of personal details [age, ethnicity, years of schooling, weight and height, Body Mass Index (BMI), physical activity (how many times a week they engaged in physical activity) and smoking history] and health background (information about the course of the PC, its advancement, staging and grading [the Tumor, Node, Metastasis staging system (TNM) [77], the Gleason Score (GS) [78], the Grade Group according to the International Society of Urological Pathology classification (GG) [79], timing of prostatectomy, adjuvant therapy and comorbidities, that could impact cognitive function such as hypertension, diabetes, myocardial infarction or stroke, as well as data on medications used). Lastly, we conducted a survey to assess erectile function—using the International Index of Erectile Function, 5-item version (IIEF-5) [80]—and urinary incontinence—Polish version of The International Consultation on Incontinence Questionnaire Short Form (ICIQ-UI SF)—before and after surgery [81].

IIEF-5—the scale contains five questions related to erection. The subject must select the most fitting answer from six options (0–5) for each question. A higher score indicates a better quality of the erection.

ICIQ-UI SF—this questionnaire briefly assesses how often urinary incontinence occurs, its severity, its impact on quality of life. More severe symptoms correspond to a higher score.

Biochemical parameters were performed from peripheral blood sample. Blood samples were taken in tube without anticoagulant and preservative. Then, serum was separated. The following parameters were determined in each patient: serum testosterone and total prostate-specific antigen (PSA) levels, C-reactive protein (CRP), pNF-H, BDNF, NSP, and Il-6. They were determined by an experienced laboratory scientist by enzyme-linked immunosorbent assay (ELISA) kits using a spectrophotometer, in accordance with manufacturers’ instructions.

Neuropsychological evaluation of cognitive functions included several tests from the “Neurotest” computer battery. In this study, we perform the test as follows:

### 4.1. Simple Reaction Time (SRT)

The SRT measures psychomotor speed and overall alertness. In this test, the participant’s task is to press the button after seeing a green circle appearing on the computer screen. Participants are told to react instantly. The metrics collected include the number of correct answers and the average response time in milliseconds.

### 4.2. Verbal Memory Test (VMT)

Five consecutive stages make up the test. Five times, the researcher reads a list of ten words. Following every repetition, participants recount as many words as they can remember from the presented list, disregarding order. The word groups remained identical across the five repetitions. The number of correctly repeated words, the number of words outside the list (intrusions), and the number of repetitions are counted (for each attempt). This task evaluates the efficiency of working memory (first attempt—VM1), short-term memory (subsequent attempts—VM2–VM5), and learning (improved results with repetition).

### 4.3. Verbal Memory Deferred Test (VMDT)

Participants, after a 20 min delay, recall the ten words in any order, without the researcher reading them aloud beforehand. This test examines deferred memory.

### 4.4. GoNoGo Test

The primary purpose of the GoNoGo test is to assess inhibitory control and executive functions. The test uses two reactions, symbolically labeled “Go” and “NoGo”. The task involves responding to green squares (“Go”) with a keypress, and withholding responses to blue squares (“NoGo”). The stimuli are presented in a random manner (75 green and 25 blue squares). The study analyzes the count and response times (ms) for correct “Go” and “NoGo” reactions.

### 4.5. Visual Working Memory Test (VWMT)

VWMT assesses visuospatial memory. The test begins with seven covered playing cards shown on the computer screen. Next, each card is revealed for two seconds before being covered again. The seven cards differ in terms of numbers and figures, as in a standard deck of playing cards. The participant’s task is to memorize the card layout. Following the presentation of all seven cards, a previously shown card appears at the screen’s top. Participants should point to where the card was previously located. The number of correctly and incorrectly indicated locations and time of reaction are recorded.

The Bioethical Commission of the Nicolaus Copernicus University, Collegium Medicum in Bydgoszcz approved the study (Approval No 508/2017). Study procedures fully complied with the Declaration of Helsinki.

### 4.6. Statistical Analysis

Statistical analysis was performed using Statistica 13.1 software. The Shapiro–Wilk test was initially employed to assess the normality of variable distributions. As the data did not follow a normal distribution, nonparametric tests were used. The Mann–Whitney U test was applied to evaluate statistically significant differences between two groups, while the Kruskal–Wallis ANOVA was used for comparisons involving more than two groups. Correlation analyses were conducted using the Spearman rank-order correlation (Spearman’s R) to determine the strength and significance of associations between variables.

## 5. Conclusions

Existing literature suggests that IL-6, NSP, pNF-H, and BDNF may serve as important biochemical markers of cognitive function in individuals with cancer. However, our study identified only limited associations. Specifically, higher levels of BDNF and NSP were significantly related to better performance on the delayed memory test. These associations, though statistically significant, were weak and require validation in larger, longitudinal cohorts. Overall, our findings underscore the complexity of the relationship between biochemical markers and cognitive functions, which may not be immediately evident. Further research is necessary to elucidate these associations and better understand the underlying mechanisms.

The existing literature indicates that IL-6, NSP, pNF-H, and BDNF may serve as relevant biochemical markers of cognitive function in individuals with cancer. In our study, however, we identified only limited associations. Specifically, higher levels of BDNF and NSP were significantly related to better performance on the delayed memory test. These associations, though statistically significant, were weak and require validation in larger, longitudinal cohorts, suggesting a potential role of these markers in delayed memory impairment. Overall, our findings underscore the complexity of the relationship between biochemical markers and cognitive function, which may not be immediately apparent. Further research is needed to clarify these associations and to elucidate the underlying mechanisms.

### Limitations

This study has several limitations. The small sample size and variability in the timing of post-surgical evaluations (the median interval of 1.5 years after prostatectomy) reduce the generalizability of the findings. Additionally, the use of a single-point biochemical assessment may not fully capture dynamic changes over time and cannot be considered indicative of cognitive impairment; repeated measurements could provide more insight into temporal patterns and correlations. The cross-sectional approach is a particular problem for a marker like pNF-H, which reflects acute axonal injury. To draw more definitive conclusions, future studies should include larger, more homogeneous cohorts and incorporate more standardized, longitudinal designs with serial biomarker evaluations that would better capture dynamic changes and their temporal correlation with cognitive function.

## Figures and Tables

**Table 1:** Demographic, clinical, and cognitive data.

Parameter		Median (Q25–Q75)
Age [y]		66.0 (60.0–70.0)
BMI		27.1 (25.1–29.7)
Years from surgery [y]		1.5 (1.0–3.2)
GRADE group [n]	1	n = 58
	2	n = 32
	3	n = 4
	4	n = 3
	5	n = 3
Diabetes (*n*, %)		13 (13%)
Hypertension (*n*, %)		56 (56%)
MI (*n*, %)		9 (9%)
Stroke (*n*, %)		7 (7%)
Education	Basic (*n*, %)	5 (5%)
	Vocational (*n*, %)	28 (28%)
	Secondary (*n*, %)	34 (34%)
	Higher (*n*, %)	33 (33%)
BDNF [ng/mL]		0.79 (0.27–1.34)
Il-6 [pg/mL]		3.26 (2.09–9.11)
NSP [ng/mL]		0.50 (0.43–0.64)
pNf-H [pg/mL]		50.2 (48.6–52.7)
SRT [n]		25.0 (25.0–25.0)
SRT RT [ms]		280.4 (234.3–319.1)
VM_1 [n]		5.0 (4.0–7.0)
VM_ 2 [n]		7.0 (6.0–8.0)
VM_3 [n]		7.0 (7.0–8.0)
VM_4 [n]		8.0 (7.0–10.0)
VM_5 [n]		8.0 (7.0–10.0)
VMDT [n]		6.0 (5.0–7.0)
GoNoGo N correct Go [n]		74.0 (73.0–75.0)
GoNoGo RT [ms]		365.8 (323.6–414.3)
GoNoGo N non correct No Go [n]		5.0 (2.0–8.0)
VWMT [n]		5.0 (3.0–6.5)
VWMT RT [ms]		3150.0 (2570.0–4441.0)

Data is presented as medians and 25th and 75th quartiles or number. Abbreviations: BMI, body mass index; Grade Group, grade of cancer advancement according to the International Society of Urological Pathology classification; BDNF, brain-derived neurotrophic factor; Il-6, interleukin 6; NSP, neuroserpin; pNf-H, phosphorylated neurofilament heavy chain; SRT, simple reaction time test; RT, reaction time; VM 1–5, verbal memory trials 1, 2, 3, 4, 5; VMDT, verbal memory delayed test; GoNoGo, GoNoGo test—correct Go answers, reaction time, incorrect NoGo answers; VWMT, visual working memory test—number of correct answers, correct answers reaction time.

**Table 2:** Analysis of serum concentrations of selected proteins in subgroups stratified by smoking status, physical activity, and body mass index.

	Non-Smokers	ACTIVE SMOKERS	*p*	Obesity	Overweight	Normal Weight	*p*	No Physical Activity	Physical Activity	*p*
**BDNF [ng/mL]**	0.91 (0.38–1.39) n = 62	0.24 (0.10–0.67) n = 10	0.08	1.05 (0.25–1.33) n = 15	0.84 (0.38–1.40) n = 40	0.44 (0.16–1.34) n = 20	0.47	0.56 (0.17–1.33) n = 23	0.84 (0.33–1.42) n = 52	0.51
**IL-6 [pg/mL]**	3.18 (1.95–5.1) n = 66	3.34 (2.36–4.91) n = 11	0.7	5.1 (1.91–16.0) n = 18	2.8 (2.1–4.6) n = 42	3.42 (2.19–7.58) n = 20	0.59	2.8 (1.95–10.7) n = 25	3.3 (2.16–6.5) n = 55	0.81
**NSP** **[ng/mL]**	0.51 (0.43–0.70) n = 67	0.49 (0.44–0.60) n = 11	0.87	0.45 (0.40–0.72) n = 18	0.53 (0.44–0.72) n = 43	0.5 (0.43–0.59) n = 20	0.47	0.47 (0.43–0.63) n = 25	0.53 (0.42–0.66) n = 56	0.75
**pNf-H** **[pg/mL]**	50 (48.6–51.7) n = 62	53 (48.1–86.3) n = 10	0.3	50.8 (48.7–52.1) n = 15	49.4 (47.5–53.4) n = 40	50.5 (49.5–57.4) n = 20	0.44	50.2 (48.1–52.7) n = 23	50.1 (48.6–52.6) n = 52	0.84

Data is presented as medians and 25th and 75th quartiles or number. Abbreviations: BDNF, brain-derived neurotrophic factor; Il-6, interleukin 6; NSP, neuroserpin; pNf-H, phosphorylated neurofilament heavy chain. We classified as obese those with body mass index > −30 kg/m^2^, overweight 25–29.9 kg/m^2^ and normal weight 18.5–24.9 kg/m^2^. We classified as physically active those who consciously engaged in physical activity a minimum of three times per week.

**Table 3:** R-Spearman correlations of cognitive test results and biochemical levels.

	BDNF [ng/mL] (n = 74)	IL 6 [pg/mL] (n = 79)	NSP [ng/mL] (n = 80)	Nfilament H [pg/mL] (n = 74)
Age [y]	r = −0.065	r = 0.115	r = −0.225; *p* = 0.04	r = 0.029
BMI	r = 0.040	r = 0.061	r = −0.020	r = −0.002
SRT [n]	r = −0.161	r = 0.095	r = 0.172	r = 0.088
SRT RT [ms]	r = −0.089	r = −0.106	r = 0.004	r = −0.045
VM_1 [n]	r = 0.175	r = −0.044	r = 0.028	r = −0.128
VM_2 [n]	r = 0.176	r = 0.087	r = 0.184	r = −0.204
VM_3 [n]	r = 0.106	r = 0.210	r = 0.156	r = −0.154
VM_4 [n]	r = 0.137	r = 0.219	r = 0.203	r = −0.172
VM_5 [n]	r = 0.129	r = 0.216	r = 0.197	r = −0.179
VMDT [n]	r = 0.231; *p* = 0.047	r = 0.101	r = 0.270; *p* = 0.015	r = −0.084
GoNoGo N correct [n]	r = −0.082	r = 0.088	r = 0.04	r = 0.183
GoNoGo RT [ms]	r = −0.064	r = −0.027	r = 0.021	r = 0.089
GoNoGo N non correct No Go [n]	r = 0.205	r = −0.025	r = −0.026	r = −0.181
VWMT [n]	r = 0.032	r = 0.024	r = −0.067	r = 0.057
VWMT RT [ms]	r = −0.016	r = 0.101	r = −0.208	r = 0.073

R-Spearman correlations results. Significant results are highlighted with red. Abbreviations: BMI, body mass index; SRT, simple reaction time test; RT, reaction time; VM 1–5, verbal memory trials 1, 2, 3, 4, 5; VMDT, verbal memory delayed test; GoNoGo, GoNoGo test—correct Go answers, reaction time, incorrect NoGo answers; VWMT, visual working memory test—number of correct answers, correct answers reaction time.

**Table 4:** R-Spearman correlations of VMDT with other cognitive tests results.

n = 94	VMDT_Number Correct	VMDT_Intrusion	VMDT_Perseveration
Age [y]	r = −0.192;	r = −0.055	r = −0.133
BMI	r = −0.037	r = 0.030	r = 0.022
SRT [n]	r = −0.021	r = 0.047	r = 0.027
SRT RT [ms]	r = −0.377; *p* = 0.0001	r = −0.266; *p* = 0.008	r = −0.160
VM_1 [n]	r = 0.307; *p* = 0.002	r = −0.042	r = −175
VM_2 [n]	r = 0.306; *p* = 0.002	r = −0.068	r = −0.135;
VM_3 [n]	r = 0.404; *p* = 0.00004	r = −0.235; *p* = 0.021	r = −0.172
VM_4 [n]	r = 0.573; *p* < 0.0000001	r = −0.220; *p* = 0.031	r = −0.006
VM_5 [n]	0.579; *p* < 0.0000001	r = −0.255; *p* = 0.026	r = −0.002
VM_intrusions [n]	r = 0.027	r = 0.052	r = −0.044
VM_perseverations [n]	r = 0.199	r = 0.196	r = 0.509; *p* < 0.0000001
GoNoGo N correct [n]	r = 0.061	r = 0.133	r = 0.172
GoNoGo RT [ms]	r = −0.412; *p* = 0.00003;	r = 0.015	r = −0.208; *p* = 0.041
GoNoGo N non correct No Go [n]	0.137	r = −0.013	r = 0.143
VWMT RT [ms]	−0.315; *p* = 0.001	r = −0.021	r = −0.214; *p* = 0.036
VWMT [n]	0.199; *p* = 0.05	r = 0.102	r = −0.092

R-Spearman correlations results. Significant results are highlighted with red. Abbreviations: BMI, body mass index; SRT, simple reaction time test; RT, reaction time; VM 1–5, verbal memory trials 1, 2, 3, 4, 5; VMDT, verbal memory delayed test; GoNoGo, GoNoGo test—correct Go answers, reaction time, incorrect NoGo answers; VWMT, visual working memory test—number of correct answers, correct answers reaction time.

## Data Availability

The original contributions presented in this study are included in the article. Further inquiries can be directed to the corresponding author.

## Abbreviations

The following abbreviations are used in this manuscript:

Aβ	beta-amyloid
AD	Alzheimer’s disease
BDNF	brain-derived neurotrophic factor
BMI	body mass index
CNS	central nervous system
CRP	C-reactive protein
GG	the Grade Group according to the International Society of Urological Pathology classification
GNG	GoNoGo Test
GS	the Gleason Score
ICIQ-UI SF	The International Consultation on Incontinence Questionnaire Short Form
IIEF-5	the International Index of Erectile Function, 5-item version
Il-6	interleukin 6
NSP	neuroserpin
PC	prostate cancer
pNF-H	the high-molecular-weight neurofilament heavy subunit
PSA	prostate-specific antigen
SRT	Simple reaction time
TNM	the Tumor, Node, Metastasis staging system
VMDT	Verbal memory deferred test
VMT	Verbal memory test
VWMT	Visual working memory test
